# Midpalatal Suture CBCT Image Quantitive Characteristics Analysis Based on Machine Learning Algorithm Construction and Optimization

**DOI:** 10.3390/bioengineering9070316

**Published:** 2022-07-14

**Authors:** Lu Gao, Zhiyu Chen, Lin Zang, Zhipeng Sun, Qing Wang, Guoxia Yu

**Affiliations:** 1 Department of Stomatology, Beijing Children’s Hospital, Capital Medical University, National Center for Children’s Health, Beijing 100045, China; gaolu_bch@outlook.com; 2 School of Software Engineering, North University of China, Taiyuan 030051, China; SF201304@st.nuc.edu.cn; 3 Pharmacovigilance Research Center for Information Technology and Data Science, Cross-Strait Tsinghua Research Institute, Xiamen 361000, China; 18010020958@189.cn; 4 National Engineering Laboratory for Digital and Material Technology of Stomatology, Beijing Key Laboratory of Digital Stomatology, Department of Oral and Maxillofacial Radiology, Peking University School and Hospital of Stomatology, Beijing 100081, China; sunzhipeng@bjmu.edu.cn; 5 Department of Automation, Tsinghua University, Beijing 100084, China; 6 National Clinical Research Center for Respiratory Diseases, Beijing Children’s Hospital, Capital Medical University, National Center for Children’s Health, Beijing 100045, China

**Keywords:** maxillary transverse deficiency, children and adolescents’ developmental status, midpalatal suture maturation and ossification status, image fusion, computer-aided diagnosis, deep convolutional neural network

## Abstract

Background: Midpalatal suture maturation and ossification status is the basis for appraising maxillary transverse developmental status. Methods: We established a midpalatal suture cone-beam computed tomography (CBCT) normalized database of the growth population, including 1006 CBCT files from 690 participants younger than 24 years old. The midpalatal suture region of interest (ROI) labeling was completed by two experienced clinical experts. The CBCT image fusion algorithm and image texture feature analysis algorithm were constructed and optimized. The age range prediction convolutional neural network (CNN) was conducted and tested. Results: The midpalatal suture fusion images contain complete semantic information for appraising midpalatal suture maturation and ossification status during the fast growth and development period. Correlation and homogeneity are the two texture features with the strongest relevance to chronological age. The overall performance of the age range prediction CNN model is satisfactory, especially in the 4 to 10 years range and the 17 to 23 years range, while for the 13 to 14 years range, the model performance is compromised. Conclusions: The image fusion algorithm can help show the overall perspective of the midpalatal suture in one fused image effectively. Furthermore, clinical decisions for maxillary transverse deficiency should be appraised by midpalatal suture image features directly rather than by age, especially in the 13 to 14 years range.

## 1. Introduction

Maxillary deficiency is a type of craniofacial malformation with a high population incidence exceeding 20% of the global population [1]. Maxillary transverse deficiency plays an important role in maxillary deficiency and results in various malocclusions, including posterior crossbite, dentition crowding, and can even lead to obstructive sleep apnea, etc. [1,2,3,4]. Moreover, dentofacial deformities, including craniosynostosis and cleft lip/palate, can also be accompanied by maxillary transverse deficiency [1,5]. Maxillary transverse deficiency impairs patients’ oral and maxillofacial development and function, facial aesthetics, and even long-term health and life quality [1,6].

Rapid maxillary expansion (RME), the routine treatment procedure to correct maxillary transverse deficiency, was created by Angell and then developed by Haas and others [7,8,9,10]. Currently, RME methods consist of tooth-borne expansion, micro-implant-assisted expansion, and surgically-assisted expansion [1,11]. The timing and treatment-induced trauma of various RME methods are distinctly different. The treatment timing is vital in determining curative effects and the severity of side effects for each RME method [12]. Expansion during inappropriate timing can cause unnecessary trauma [12], as well as increasing side effects [13,14,15,16]. An accurate appraisal of maxillary transverse developmental status is critical to clarify the appropriate timing for different treatment methods [1]. 

The midpalatal suture is the main site of maxillary growth and development and also the main resistance site for RME [17]. The accurate and efficient appraisal of midpalatal suture maturation and ossification status is the basis for appraisal of maxillary transverse developmental status. 

Midpalatal suture is known for its narrow and complex anatomical morphological characteristics [1]. Several appraisal methods for midpalatal suture maturation and ossification status have been reported, such as histological methods and imaging methods, including occlusal radiography, ultrasonography, and computed tomography (CT), especially cone-beam CT (CBCT) [18,19,20,21,22,23]. The histological result is the golden standard but is not practical for in vivo routine clinical examination. Occlusal radiography was used in some studies but was gradually replaced by CT due to the superimposition of adjacent structures [24]. Compared with multislice spiral CT, CBCT also provides accurate three-dimensional visualization with better skeletal contrast resolution, lower radiation exposure, and lower cost [24,25]. 

Up to now, the existing CBCT studies for midpalatal suture maturation and ossification are mostly based on single-image sections, and their qualitative or quantitive analysis were carried out by humans [22,26], and therefore face obvious challenges which will lead to the loss of large amounts of valuable image information, high technical sensitivity as well as low feasibility and simplicity [22,27,28,29,30]. Evidence provided by the current CBCT appraisal methods is not qualified enough for routine clinical use [28,29,30,31]; thus, a methodological improvement in utilizing comprehensive image information to provide more reliable evidence is mainly needed [22,26].

To address these present challenges, it is necessary and essential to utilize image information comprehensively. Image fusion based on computer vision technology will help extract multi-section images’ information to the maximum extent, reduce interference, and then synthesize high-quality fusion images so as to improve image data utilization and reliability [32]. Image fusion is suitable for the comprehensive and high-quality extraction of image information from complex anatomical structures. It has been applied in craniocerebral hemorrhage and tumor, liver injury, etc., playing an important role in disease identification and diagnosis [33,34,35] and has started to be applied in oral and maxillofacial diseases, including head and neck tumor and temporal–mandibular joint diseases [36,37].

Intelligent image analysis studies in the field of oral and maxillofacial growth and development are in the early stage of exploration [38]. Chen Y. et al. carried out preliminary intelligent image analysis using CBCT axial position images based on small sample data, and their results showed the application potential of intelligent image analysis in midpalatal suture maturation and ossification status [39]. However, up to now, the image features taken from the single-section images of large field CBCT have led to a disturbance of other anatomic regions and insufficient focus on the midpalatal suture region itself. Moreover, the sample sizes of previous studies need to be enlarged.

In this study, we will establish a midpalatal suture CBCT normalized database of the growth population, innovatively extract and screen out key quantitative image characteristics comprehensively by image fusion, and then analyze the correlation between quantitative image characteristics of the midpalatal suture and chronological age.

Compared with previous studies that extracted and analyzed midpalatal suture image characteristics through a single image section, we designed an image fusion algorithm to utilize multi-slice valuable image information in CBCT. This image fusion algorithm avoids the influence of CBCT examination orientation and the convex palatal vault, therefore helping to show the overall perspective of the midpalatal suture in one fused image [40,41,42]. Furthermore, structure labeling by clinical experts will improve the proportion of midpalatal sutures in the final images.

The remainder of this article is organized as follows: the automated processing techniques of CBCT, midpalatal suture region image fusion method, and the chronological age range prediction model are all covered in Section 2; the performance of the proposed methods is evaluated in Section 3. Finally, we present some discussions in Section 4 and conclude this article in Section 5.

## 2. Materials and Methods

### 2.1. Midpalatal Suture CBCT Normalized Database of Growth Population

#### 2.1.1. Samples

The study was conducted in accordance with the Declaration of Helsinki, and the protocol was approved by the Ethics Committee of the Peking University Hospital of Stomatology Institutional Review Board (PKUSSIRB-202163037).

The sample collection was carried out at the Peking University Hospital of Stomatology. CBCT from patients younger than 24 years old that undergone single or multiple CBCT examinations in the Department of oral and maxillofacial radiology according to diagnosis or treatment needs (1 January 2015 to 31 December 2020) were screened. The examination field should include the supra-orbital arch (upper boundary) and the lower margin of the fourth cervical vertebra (lower boundary), and the examination interval for the same participant should be longer than 1 month. The exclusion criteria are shown in Table 1. The gender and clinical departments of the participants were not limited.

#### 2.1.2. CBCT Examination

**Figure 1:**
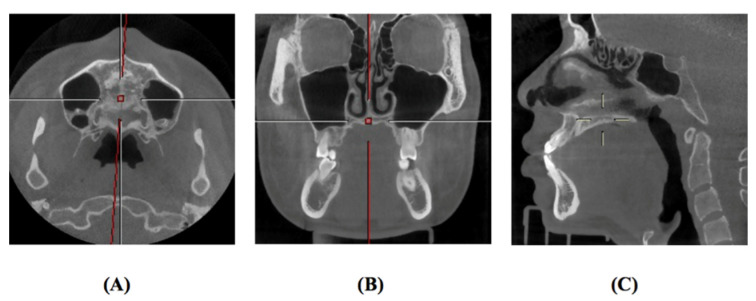
Head orientation in CBCT examination. (**A**): Axial view; (**B**): Coronal view; (**C**): Sagittal view.

CBCT images were taken with NewTom VGi (Quantitative Radiology, Verona, Italy), at 2.81 mA, 110 kV, 3.6-s exposure, and a 15 × 15 cm field of view, with an axial slice thickness of 0.3 mm, and isotropic voxels (Figure 1). The participants sat upright with a natural head position and jaws immobilized using a chin holder, keeping the Frankfort plane horizontal to the ground. The teeth were occluded at the intercuspal position, with facial muscles relaxed.

### 2.2. Region of Interest Labeling in Midpalatal Suture CBCT Images

**Figure 2:**
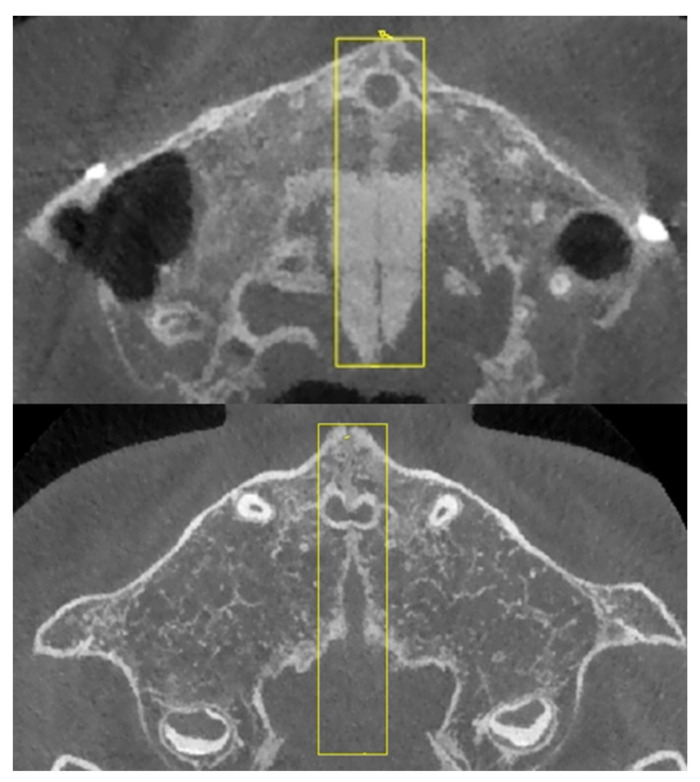
Labeling sample of the midpalatal suture region.

The region of interest (ROI) labeling was completed by two experienced clinical experts. The upper and lower boundaries of the CBCT axial sections for each CBCT file were located by Dolphin Imaging software (11.8, Oakdale, CA, USA) and recorded by Microsoft Excel software (2203, Redmond, WA, USA). The anterior and posterior boundaries of the CBCT axial sections for each CBCT file were located by MicroDicom DICOM viewer software (2022.1, Sofia, Bulgaria) and recorded by Colabeler software (2.0.4, Hangzhou, China) (Figure 2).

The upper boundary of the CBCT axial sections is the upper margin of the palatal vault, the lower boundary is the apical point of the upper central incisors (choose the higher one when the two apical points are in different sections), the anterior boundary is the most anterior point of the midpalatal suture on the maxilla, and the posterior boundary is the most posterior point of the midpalatal suture on the palatine bone.

### 2.3. Image Analysis Algorithm

The algorithm in this study consists of two parts: the midpalatal suture CBCT image fusion algorithm (introduced in Section 2.3) and the image texture feature analysis algorithm (introduced in Section 2.4). 

As the midpalatal suture image is complicated, it is difficult to be obtained through single-section image analysis. In addition, the proportion of midpalatal suture in the total CBCT field is small; thus, more noises will arise from other regions apart from the midpalatal suture. Therefore, the raw images cannot be applied to appraise the maturation and ossification status effectively or be used to train a convolutional neural network (CNN) [43]. Therefore, we proposed a CBCT image fusion algorithm, which includes three parts: image processing, image fusion, and fused image optimization.

#### 2.3.1. Image Processing

The CBCT files were read and converted into three-dimensional gray matrixes and then converted into a series of axial images of 512 × 512 resolution. The midpalatal suture normalized ROI of 50 × 200 resolution were extracted.

#### 2.3.2. Image Fusion

The fusion weights were calculated and adjusted by combining the existing pixel-level image fusion algorithm with the characteristics of the midpalatal suture region. Image fusion was carried out in every two sections of midpalatal suture multi-slice ROI images for each CBCT file until all of the images were fused into one overall midpalatal suture image. The pixel value of each point in the fused image was calculated by the following formula:

(1) $$P_{\mathrm{ij}}=A_{\mathrm{ij}}*\left(1+\frac{A_{\mathrm{ij}}-e}{255+d}\right)$$

$A_{\mathrm{ij}}$ refers to the average gray scale value of point (i, j) in each of the two images that need to be fused, e refers to the total average gray scale of the images that need to be fused, and d refers to the adjustment factor based on the maximum gray scale difference of the images that need to be fused. 

It can be predicted that if all of the images are fused directly, all of the pixels will approach the average gray level, resulting in blurred fused images. Therefore, we performed the weighted fusion of images in pairs and then continued to fuse the fused images in pairs until all of the images were fused into one. The computational complexity of the image fusion algorithm is O(nlogn), since the structure of the image fusion algorithm is merging.

#### 2.3.3. Fused Image Optimization

During the image fusion process, we used the convolution operator to optimize the fused image so as to improve the clarity of the midpalatal suture. The operator weight was adjusted according to the image fusion result to make the image textures clearer.

### 2.4. Image Texture Feature Analysis Algorithm

The image texture feature analysis was then conducted to find the correlation between the midpalatal suture CBCT image texture features and chronological age. Compared with CNN, the image texture features training is more intuitive. The preliminary texture features analysis can also provide evidence for the effectiveness of CNN training since the CNN lacks interpretability.

Six typical features, including correlation, contrast, homogeneity, dissimilarity, angular second moment (ASM), and energy, were analyzed (Table 2). 

The image texture features were extracted by Scikit-Image. Then, scatter diagrams of all samples were drawn by pyplot, in which chronological age was taken as an independent variable and image texture feature value was taken as a dependent variable. Correlations between image texture features with chronological age were evaluated to find out if they are suitable to appraise midpalatal suture maturation and ossification status.

### 2.5. Age Range Prediction of Midpalatal Suture CBCT Image Features

The age range prediction CNN model was carried out to further clear the prediction efficiency of midpalatal suture maturation status image features for chronological age.

#### 2.5.1. Datasets and Labels

Five age ranges were classified and labeled: 4 to 10 years old labeled as 0, 11 to 12 years old labeled as 1, 13 to 14 years old labeled as 2, 15 to 16 years old labeled as 3, and 17 to 23 years old labeled as 4. In addition, the data were expanded through random translation, tilt, contrast and brightness adjustment, clip in small amplitude, and horizontal mirroring. The finally adjusted images were normalized into 50 × 200 pixels.

(1) Validation set: Out of the total samples, 10 typical samples were selected from each age range, and these 50 images were used as the validation set.
(2) Test set: Out of the total samples, 20 typical samples were selected from each age range, and these 100 images were used as the test set.
(3) Training set: Out of the total samples, the remaining 856 samples, apart from those used in the validation set and test set, were used as the training set.

**Figure 3:**
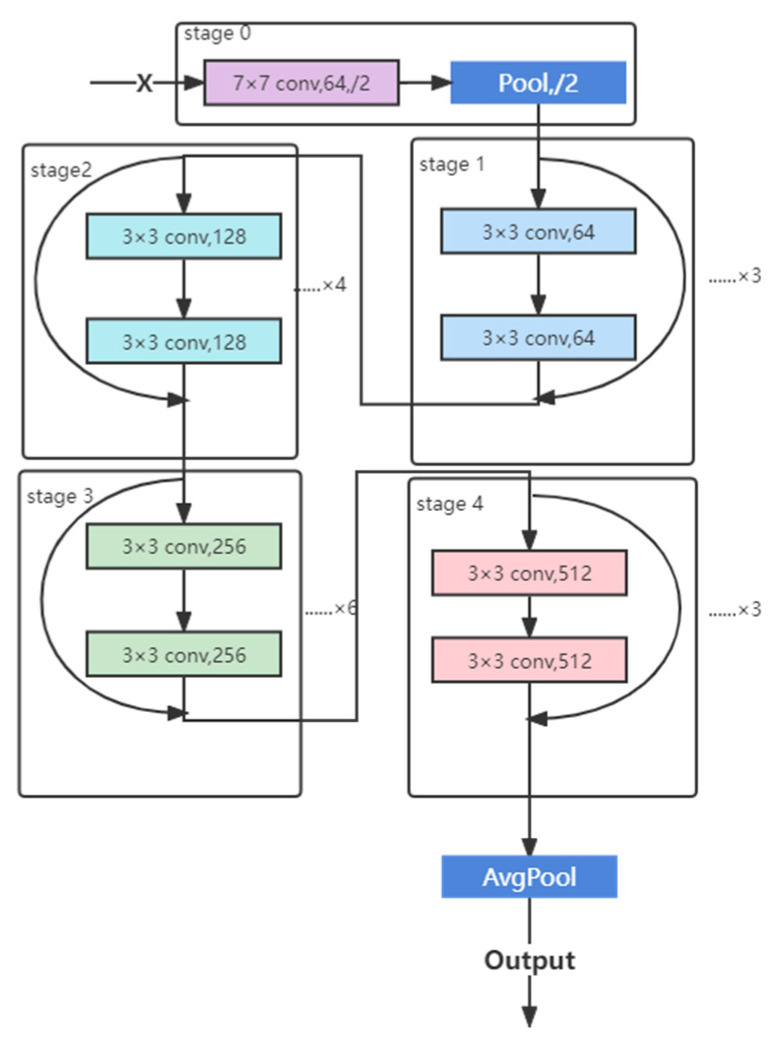
ResNet structure.

The optimized deep residual network (ResNet) 50 model CNN was used to conduct the chronological age range prediction (Figure 3). Age range prediction by midpalatal suture image is a multi-classification task. The Softmax function was used in the output layer to make the total probability of five age ranges equal to 1. Then the cross-entropy loss function was used to quantify the error between the model outputs and labels. Grad-CAM was applied to generate heat maps for model prediction. The redder the color is, the more dependent the model is on the image features of this region.

#### 2.5.2. CNN

As the most widely used deep learning method, CNN was used in age range prediction tasks by using midpalatal suture fused images [44]. 

The CNN in our study mainly consisted of an input layer, a convolutional layer, a pooling layer, a full connected layer, and an output layer. The input was the raw image X. $X_{i}$ refers to the feature map of layer *i* ($X_{0}=X$). As the convolution layer, $X_{i}$ was generated by the following formula:

(2) $$X_{i}=f\left(X_{i-1}⊗W_{i}+b_{i}\right)$$

$W_{i}$ represents the weight vector for the convolution kernel of *i*, the ⊗ symbol represents the convolution operation between the convolution kernel and the (*i* − 1) layer image. The output of the convolution was added to $b_{i}\;$ (offset vector of layer *i*). Finally, $X_{i}$ (feature image of layer *i*) was obtained through the nonlinear excitation function f(X). 

The convolutional layer was followed by the pooling layer. The pooling layer compressed the input feature image to reduce feature dimensions, thus simplifying the complexity of the CNN calculation, while maintaining certain invariance of the feature (rotation, translation, expansion-retraction, etc.).

Essentially, the CNN was a mathematical model of mapping the original matrix to a new probability expression through data transformation or dimensionality reduction at multiple levels. After the alternating transmission of multiple convolutional layers and pooling layers, we classified the extracted image features and obtained the input-based probability distribution by the CNN relied on a fully connected network.

#### 2.5.3. Deep Residual Learning

ResNet solves the problem of difficulty in CNN model training [45] and shows excellent performance in CNN [46,47,48]. Compared with other network structures, ResNet’s learning results are more sensitive to the fluctuations of network weights and data, and it is one of the best model choices at present. The network structure in this study is the optimized ResNet50 network.

Residual blocks in ResNet are designed to learn the residuals of underlying features rather than the underlying features. In a residual block, if the learned features for input X is recorded as H(X), the expected residual F(X)=H(X) − X. In this way, the original learning feature is F(X) + X. 

**Figure 4:**
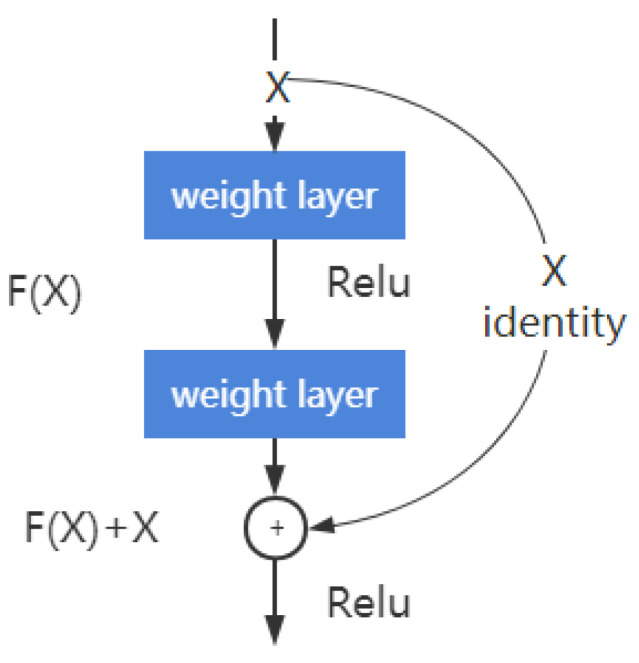
Building block of residual learning.

Deep residual learning is easier than directly learning original features. When the residual is 0, there is only identity mapping in the accumulation layer, and at least the network performance will not decline. In fact, the residual will not be 0; thus, deep residual learning will enable the accumulation layer to learn new features based on input features so as to improve performance. The residual learning process is a shortcut connection (Figure 4), which is similar to a short circuit in the electric circuit.

Intuitively, the learning content reduces residual learning. The residual is relatively small, making the learning process easy. The residual unit is expressed as the following formulas:

(3) $$y_{l}=x_{l}+F\left(x_{l},W_{l}\right)$$

(4) $$x_{l+1}=f\left(y_{l}\right)$$

$x_{l}$ and $x_{l+1}$ represent the input and output of residual unit of *l*, respectively. Each residual unit is a multi-layer structure. F, as the residual function, represents the learned residual. The $h\left(x_{l}\right)=x_{l}$ represents the identity mapping, and f is the ReLU activation function. Based on Formulas (3) and (4), the learning features from shallower layer l to deeper layer L is:

(5) $$x_{L}=x_{l}+\sum_{i=l}^{L-1}F\left(x_{i},W_{i}\right)$$

The gradient of the reverse process can be obtained by the chain rule:

(6) $$\frac{\partial loss}{\partial x_{l}}=\frac{\partial loss}{\partial x_{L}}\cdot \frac{\partial x_{L}}{\partial x_{l}}=\frac{\partial loss}{\partial x_{L}}\cdot \left(1+\frac{\partial }{\partial x_{l}}\sum_{i=l}^{L-1}F\left(x_{i},W_{i}\right)\right)$$

The first factor $\frac{\partial loss}{\partial x_{l}}$ represents the gradient of the loss function to L. The “1” in the parentheses represents that the short-circuit mechanism can spread the gradient nondestructively, while the other residual gradient needs to pass through layers with weights. The gradient is not directly transmitted.

#### 2.5.4. ResNet Structure

As shown in Figure 3, ResNet was divided into 5 stages, wherein stage 0 contains one convolution layer and one pooling layer, and stages 1 to stage 4 contain 3, 4, 6, and 3 convolution accumulation structures, respectively. Finally, the output results were converted from the average pooling layer.

#### 2.5.5. Hyperparameters Selection

In terms of hyperparameter selection, we firstly used the recognized parameters with excellent performance for model training. Then, within the specified parameter range, we used the grid search method to adjust the parameters by step. According to the performance of the saved model on the test set, the best set of hyperparameters was selected from all of the hyperparameters. The final selected hyperparameters are shown in Table 3.

#### 2.5.6. Feature-Based Visualization

The training process of CNN is generally considered a “black box”, and the model lacks intuitive interpretability [49]. Therefore, the Grad-CAM [50] method was adopted to generate heat maps according to the dependence degree of the midpalatal suture image feature region. The redder color of the region, the stronger the dependence of the model on the image feature of that region in the prediction process.

In Grad-CAM, the gradient of network back propagation was used to calculate the weight of each channel in the heat map. For the category c, the weight α of each channel was first obtained. Then the weighted sum of data from all of the channels in the feature layer A was calculated. Finally, the heat map was obtained by the ReLU activation function. The formulas are as follows:

(7) $$\alpha_{k}^{c}=\frac{1}{Z}\sum_{i}\sum_{j}\frac{\partial y^{c}}{\partial A_{ij}^{k}}$$

(8) $$L_{\mathrm{Grad}-\mathrm{CAM}}^{c}=\mathrm{ReLU}\left(\sum_{k}\alpha_{k}^{c}A^{k}\right)$$

In Formulas (7) and (8), c refers to category, $y^{c}$ refers to the score that has been forecasted by the neural network but without softmax processing. A represents the feature value of the last convolution output layer, *k* refers to the *k*-th channel of feature layer A, $A^{k}$ refers to the calculation value of the *k*-th channel in feature layer A, $A_{ij}^{k}$ refers to the calculation value of coordinate point (*i*, *j*) in the *k*-th channel of feature layer A. Z refers to the size of the feature layer (e.g., width × height). 

Each Grad-CAM heat map was superposed with the fused image for age range prediction so as to intuitively show the dependence degree of the model on that image region in the prediction process and help further evaluate the rationality of the model.

## 3. Results

### 3.1. Demographic Characteristic

The midpalatal suture CBCT normalized database with a total of 1006 CBCT files (CBCT files of females: 610, CBCT files of males: 396) was obtained from 690 participants of the growth population (female: 403, male: 287). In the database, there are 414 participants with single-time CBCT, 245 participants with two-times CBCT, 23 participants with three-times CBCT, seven participants with four-times CBCT, and one participant with five-times CBCT.

The demographic characteristics of the total 1006 CBCT files are shown in Table 4.

### 3.2. Midpalatal Suture ROI Extraction and Image Fusion Algorithm

**Figure 5:**
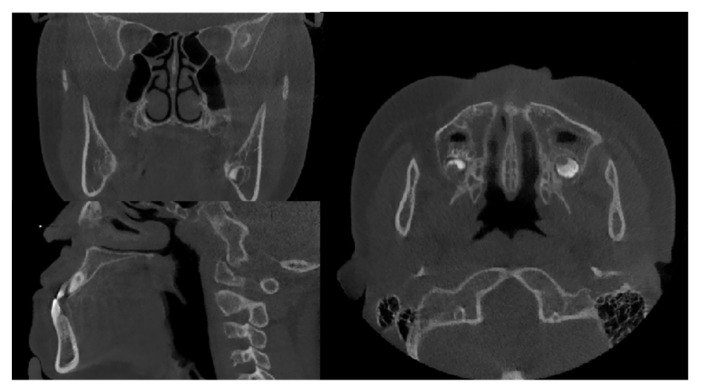
The image example of sagittal, coronal, and axial views of one CBCT file.

**Figure 6:**
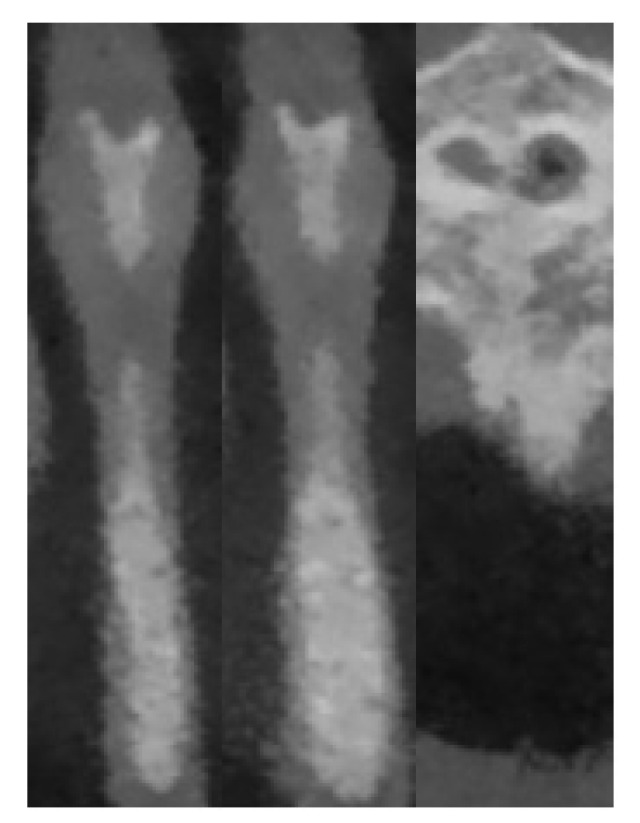
The image example of extracted region of interest (ROI) for midpalatal suture.

Figure 5 and Figure 6 show the image processing results of the midpalatal suture region. After reading, the sagittal, coronal, and axial views of each selected CBCT file contain hundreds of sections. After labeling by clinical experts, the midpalatal suture ROI images were extracted from the multi-slice axial images (Figure 6). 

**Figure 7:**
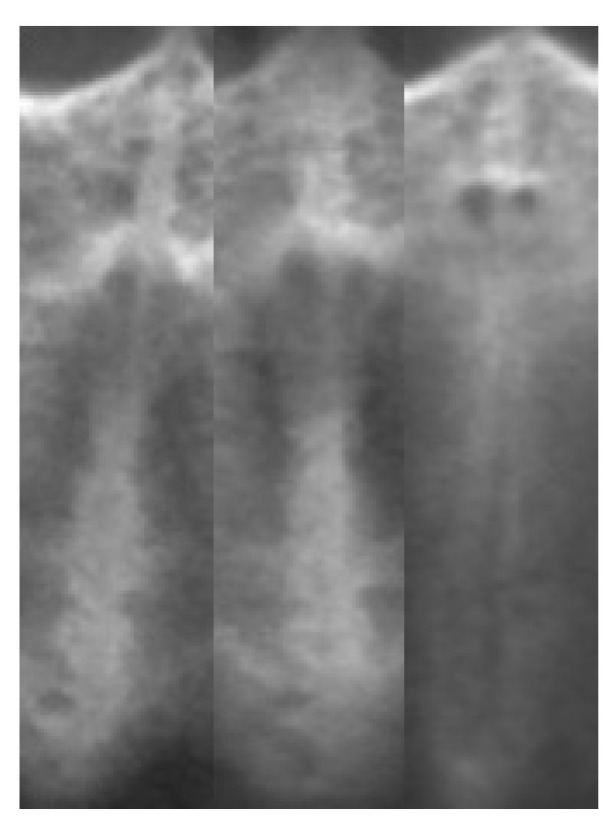
The fused image examples after direct image fusion process.

**Figure 8:**
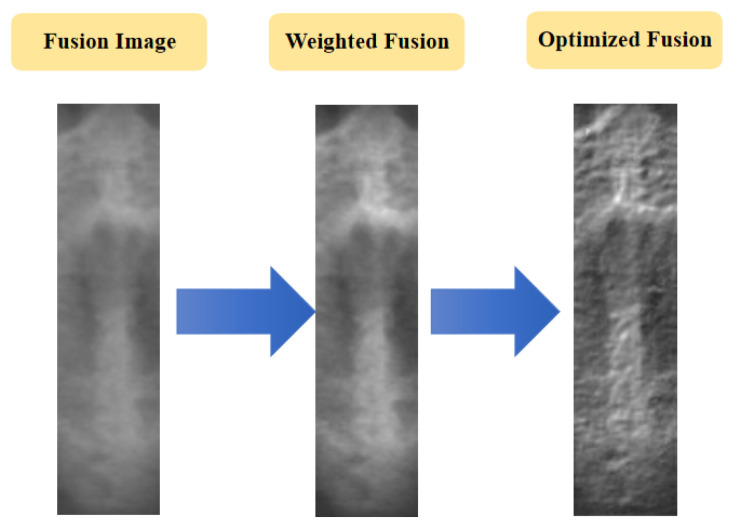
Examples of image fusion optimization process.

Then, the direct image fusion, weighted optimization, and convolution operator optimization were carried out (Figure 7 and Figure 8). The direct fusion shows poorer performance, in which the image is blurred, and the morphological characteristics of the midpalatal suture region are not clear. By adjusting the fusion weight, the image contrast increases, and the midpalatal suture structure is clearer. Furthermore, after convolution operator optimization, the fused images show clear and distinct texture, which is more conducive for clinical evaluation and subsequent model training process.

### 3.3. Image Feature Analysis 

**Figure 9:**
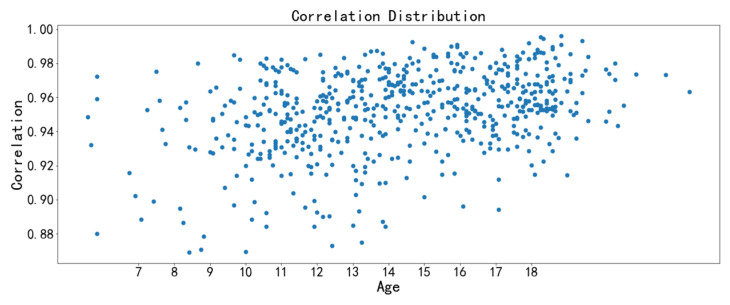
Scatter diagram between correlation with chronological age (females).

**Figure 10:**
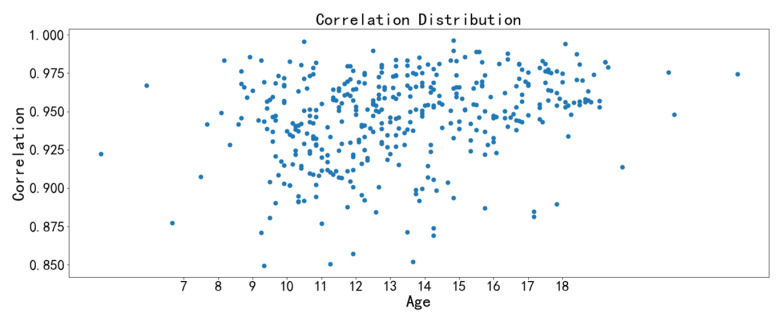
Scatter diagram between correlation with chronological age (males).

**Figure 11:**
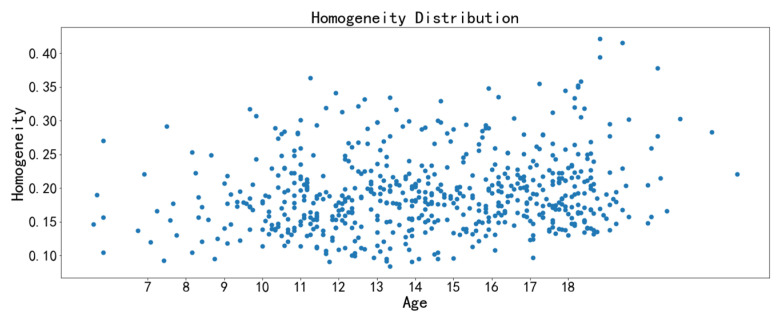
Scatter diagram between homogeneity with chronological age (females).

**Figure 12:**
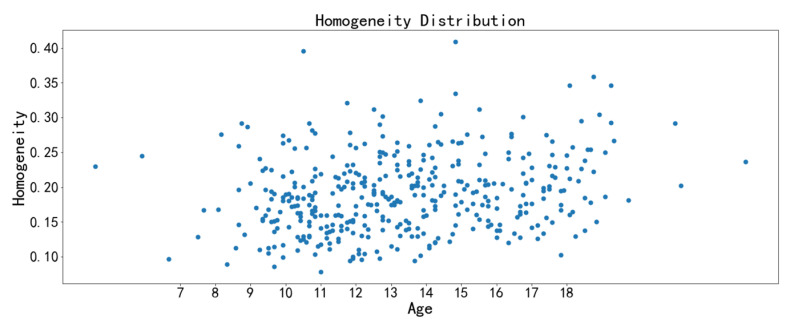
Scatter diagram between homogeneity with chronological age (males).

The image texture feature scatter diagrams show obvious positive correlations between the correlation feature with chronological age and the homogeneity feature with chronological age, respectively (Figure 9, Figure 10, Figure 11 and Figure 12). The positive correlation trends are similar among females and males.

Homogeneity is used to measure how much the local texture changes. A large value indicates that there is less change between different regions of the image texture, and the parts are more uniform. Correlation reflects the consistency of image texture. It is used to measure the similarity of spatial gray level co-occurrence matrix elements in a row or column direction. The homogeneity feature and the correlation feature both tend to increase with chronological age, which may be due to the increased maturation and ossification degree of the midpalatal suture region.

### 3.4. Age Range Prediction Model by Midpalatal Suture CBCT Image Features

#### 3.4.1. Model Evaluation

The evaluation parameters for the age range prediction model using the midpalatal suture image features include precision ratio *P*, recall ratio *R*, and the test set classification F1-score. *P* refers to the proportion of correctly classified positive samples in the positive samples determined by a classifier, and *R* refers to the proportion of correctly classified positive samples in the true positive samples. F1-score is the harmonic average of *P* and *R*, and Acc refers to the proportion of correctly identified samples in all samples. The calculation formulas are as follows:

(9) $$P_{X}=\frac{T_{\mathrm{PX}}}{T_{\mathrm{PX}}+F_{\mathrm{PX}}}$$

(10) $$R_{X}=\frac{T_{\mathrm{PX}}}{T_{\mathrm{PX}}+F_{\mathrm{NX}}}$$

(11) $$F1score_{X}=\frac{2\times P_{X}\times R_{X}}{P_{X}+R_{X}}$$

(12) $$\mathrm{Acc}=\overset{4}{\underset{X=0}{\sum }}\frac{T_{\mathrm{PX}}+T_{\mathrm{NX}}}{T_{\mathrm{PX}}+T_{\mathrm{NX}}+F_{\mathrm{PX}}+F_{\mathrm{NX}}}$$

For each age range X, $T_{\mathrm{PX}}$ refers to the number of correctly predicted samples which predicted the certain age range; $F_{\mathrm{PX}}$ refers to the number of wrongly predicted samples which predicted the certain age range; $F_{\mathrm{NX}}$ refers to the number of wrongly predicted samples which predicted to other age ranges; $T_{\mathrm{NX}}$ refers to the number of correctly predicted samples which predicted to other age ranges. 

**Figure 13:**
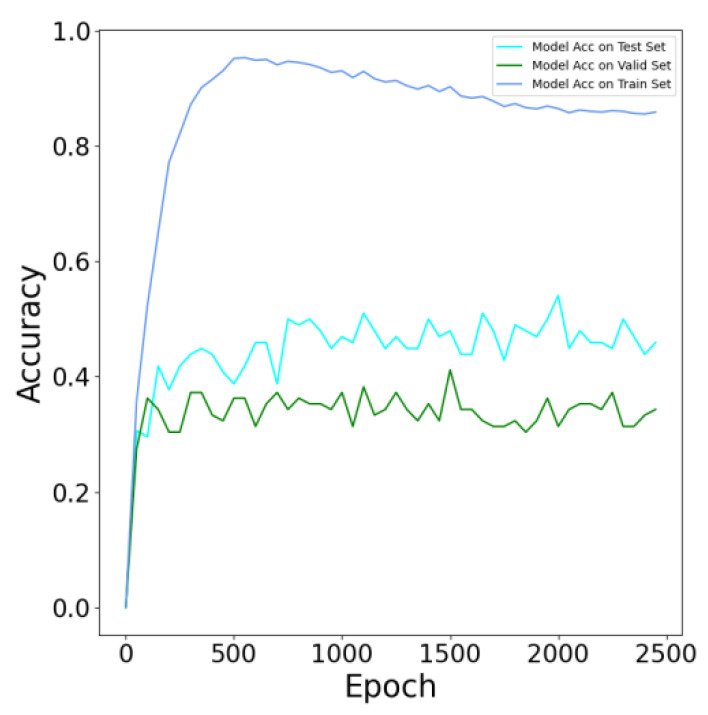
Accuracy of test set verification results of models with different f training times.

The accuracy of test set verification results of models with different f training times are shown in Figure 13, in which model accuracy reaches the maximum value in the 2000th round of training. This model was saved for further testing and analysis.

**Figure 14:**
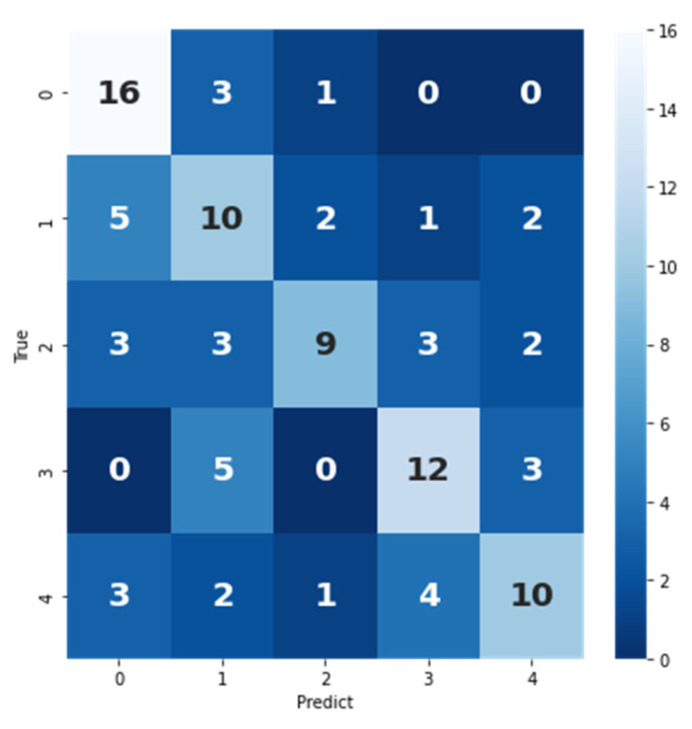
The confusion matrix of age range prediction model by midpalatal suture image features verified in the test set.

The confusion matrix of the age range prediction model by midpalatal suture image features verified in the test set is shown in Figure 14. The sum of each row represents the number of actual samples of a certain label, and the sum of each column represents the number of samples predicted as this label. *P*, *R*, F1-score, and area under curve (AUC) values of the prediction model can be calculated by the confusion matrix, and the results are shown in Table 5.

The CBCT data set in this study is self-constructed, including a total of 1006 subjects from 4 to 23 years old, while most of the subjects belong to the middle age range. For this five-category classification task, clinicians paid more attention to sensitivity, specificity, and especially the AUC value, which is 0.7532, indicating that this model has reached the clinical auxiliary level. At present, the compromised classification accuracy is limited by the data set imbalance on the one hand and optimization of the sequence fusion algorithm on the other hand. The image fusion algorithm is very important in reflecting the image characteristics of midpalatal suture maturation status for subjects of different chronological age groups. Our future work will focus on optimizing and adjusting the image fusion algorithm to further support and improve classification accuracy.

#### 3.4.2. Evaluation of Model Performance

**Figure 15:**
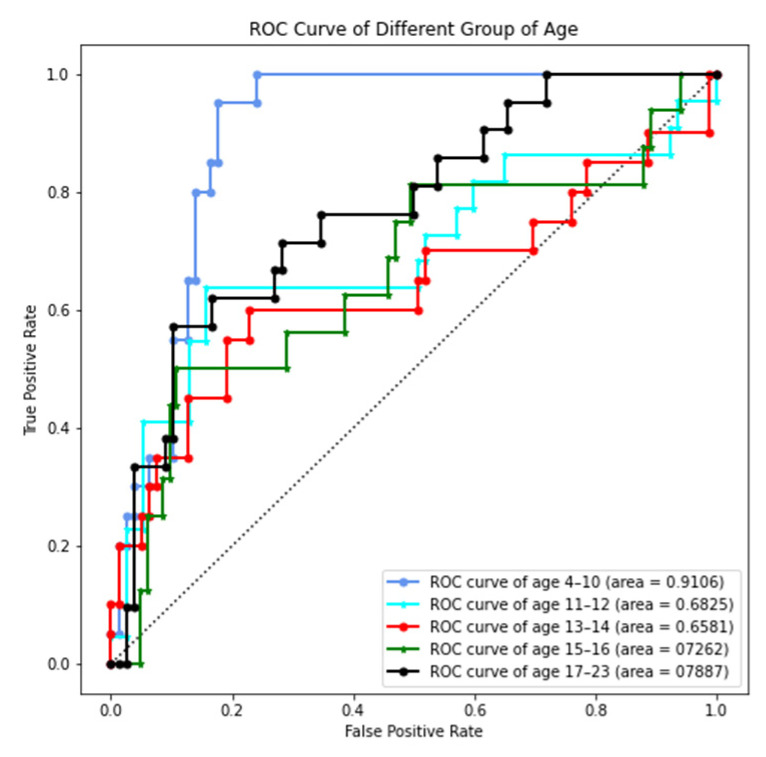
Receiver operating characteristic (ROC) curves and area under curve (AUC) values of different age ranges in the age range prediction model.

Receiver operating characteristic (ROC) curves and area under curve (AUC) values are taken to evaluate the age range prediction model (Figure 15). The true positive rate refers to the number of correctly predicted samples that are predicted to a certain age range; the false positive rate refers to the number of wrongly predicted samples that are predicted to a certain age range.

The AUC values for predicting all age ranges are above 65%, in which the AUC values of the 4 to 10 years range (0.9106) and the 17 to 23 years range (0.7887) are the two best age ranges. 

#### 3.4.3. Feature-Based Visualization

**Figure 16:**
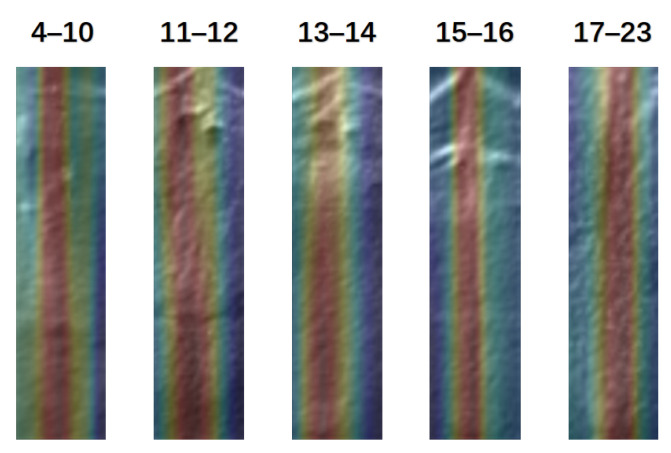
Image region heat maps based on Grad-CAM.

The image feature heat maps of the midpalatal suture region show that the redder areas are all located in the midpalatal suture (Figure 16), indicating that the image features of the midpalatal suture region have satisfactory performance in its maturation and ossification status appraisal, as well as chronological age range prediction.

## 4. Discussion

Clinical effectiveness and treatment-induced trauma of various kinds of RME methods are distinctly different. Treatment timing is vital in determining the clinical effectiveness and severity of side effects for each RME method [12]. Expansion during inappropriate timing can cause unnecessary trauma, as well as increased side effects, including periodontal attachment level loss, buccal cortical bone fenestrations, and dental root resorption [13,14,15,16]. Therefore, accurate appraisal of maxillary transverse developmental status is critical to provide evidence for the appropriate timing of different methods in maxillary transverse deficiency treatment so as to optimize the treatment strategies.

The research conception of this study is to prove the correlation between chronological age and maturation status of midpalatal suture and to provide evidence and theoretical support for our following study of establishing the staging standard of the midpalatal suture fused images. Therefore, it is necessary to prove the relationship between chronological age and maturation status of midpalatal suture through image characteristics from multiple perspectives.

### 4.1. Innovative Midpalatal Suture Image Fusion Algorithm

Ossification and maturation status of midpalatal suture is complicated. Age-related morphological changes in the midpalatal suture of human and animal specimen samples indicate that midpalatal suture can remain unfused for many years postnatal, even whole-life long period [51,52,53,54,55,56]. 

Given the histomorphological conclusions, the appraisal of midpalatal suture maturation and ossification status by “if it’s fused/obliterated” is not reliable. However, the current imaging appraisal methods, especially the CBCT appraisal methods, are mainly based on “if midpalatal suture is fused/obliterated” in a single image section and mainly through human-eye qualitative appraisal, which leads to the loss of a large amount of valuable image information, high technical sensitivity, as well as low feasibility and simplicity [22,27,28,29,30].

Therefore, quantitative imaging analysis not entirely reliant on the human eye is necessary to find more valuable information related to midpalatal suture growth and development more than its obliteration and absolute width. Image fusion has been used in this study to extract multi-section image information and then synthesize high-quality fused images. While as a widely used medical image analysis method in studies of several diseases [33,34,35], image fusion has not been applied in craniofacial growth and development studies. The combination of image fusion and craniofacial growth analysis, especially skeletal growth analysis, can help us utilize comprehensive image information of the complicated structures effectively and reliably.

### 4.2. Clinical Implications of Midpalatal Suture Image Texture Features

The correlation feature and the homogeneity feature are the two texture features with strongest relevance with chronological age for midpalatal suture fused images.

Belonging to gray-level co-occurrence matrix (GLCM) texture, the correlation feature and the homogeneity feature basically reflect the uniformity of the image texture. Higher values of correlation and homogeneity indicate that each midpalatal suture fusion image has more uniform textures [57,58]. During growth and development, the gray level of the midpalatal suture and its adjacent regions grow closer, and the image texture is more consistent, referring to the increasing maturation and ossification process. Midpalatal suture, yet not obliterated, will change a lot in morphological characteristics during this process [1,18,52,54,55]. However, much valuable image information was lost in previous studies since the image texture features are difficult to be directly recognized by human eyes. 

The positive relevance between the midpalatal suture maturation process and the overall growth status represented by chronological age indicates that even though midpalatal suture may not fuse or obliterate for many years or even during a life-long period, its maturation and ossification status experiences significant changes during the fast growth and development period, for both females and males.

### 4.3. Clinical Significance of Age Range Prediction Model by Midpalatal Suture Image Features

As mentioned above, the maturation and ossification status of a midpalatal suture experience significant changes during the fast growth and development period [1,18,52,54,55]. Our age range prediction model using the midpalatal suture image features proves that the overall prediction efficiency is satisfactory, especially for the youngest 4 to 10 years range (0.9106) and the oldest 17 to 23 years range (0.7887) (Figure 15).

Meanwhile, the prediction efficiency for the 11 to 12 years range (0.6825), the 13 to 14 years range (0.6581), and the 15 to 16 years range (0.7262) are relatively lower, especially the 13 to 14 years range (0.6581). It is in correspondence with the clinical dilemma when predicting the skeletal effectiveness of RME treatment for patients of this age range [1]. The midpalatal suture maturation and ossification process are sensitive in this age range, and individual differences are more obvious in this period than in other age ranges. If chronological age is not an efficient indicator for midpalatal suture maturation and ossification status for these patients, RME clinical effectiveness should then be appraised by midpalatal suture image features directly. Further studies should focus on identifying optimized image characteristics to appraise midpalatal suture maturation and ossification status more satisfactory than chronological age, especially for RME treatment clinical sensitive period of the 13 to 14 years range.

Compared with the previous methods that extract and analyze midpalatal suture image characteristics through a single image section, the image fusion algorithm in this study helps utilize multi-slice valuable image information to show the overall perspective of the midpalatal suture in one fused image [40,41,42]. Furthermore, structure labeling by clinical experts helps improve the proportion of midpalatal sutures in the final images. The chronological age prediction model in this study thus provides obvious indicative evidence for midpalatal suture maturation and ossification appraisal.

## 5. Conclusions

(1) We designed a midpalatal suture CBCT image fusion algorithm to utilize multi-slice valuable image information to improve the appraisal accuracy of midpalatal suture maturation and ossification status. This algorithm avoids the influence of CBCT examination orientation and the convex palatal vault, thus helping to show the overall perspective of midpalatal suture in one fused image.
(2) The correlation feature and the homogeneity feature are the two texture features with the strongest relevance to chronological age. The midpalatal suture maturation and ossification status experience significant changes during the fast growth and development period. Furthermore, the overall performance of the age range prediction CNN model by midpalatal suture image features is satisfactory, especially in the youngest 4 to 10 years range and the oldest 17 to 23 years range. While for adolescents of 13 to 14 years range, the prediction performance is compromised, indicating that RME clinical effectiveness should be appraised by midpalatal suture image features directly rather than by chronological age for this age range.
(3) There are some limitations to this study. Sample representativeness and sample size should be further improved and expanded by the addition of multicenter samples. Furthermore, the relationship between the midpalatal suture fused image features and maxillary transverse developmental status need to be further clarified to provide evidence for appraising suitable RME treatment timing.

## Figures and Tables

**Table 1:** Exclusion criteria for participants.

Exclusion Criteria
(1) History of severe systemic diseases; (2) History of cranial and maxillofacial bone fracture; (3) History of cranial and maxillofacial bone tumor; (4) History of cleft lip and/or palate; (5) History of syndromes or endocrine diseases affecting cranial and maxillofacial bone development.

**Table 2:** Description of the texture features.

Texture Feature	Description
Correlation	Correlation reflects the consistency of image texture. It is used to measure the similarity of spatial gray level co-occurrence matrix elements in row or column direction.
Homogeneity	Homogeneity is used to measure how much the local texture changes. A large value indicates that there is less change between different regions of the image texture, and the parts are more uniform.
Energy	Energy is the sum of the squares for the values of each element in the gray level co-occurrence matrix. It is a measure of the stability of the gray level change of the image texture and reflects the uniformity of the image gray level distribution and the thickness of the texture. A larger energy value indicates that the current texture is stable, with regular changes.
Contrast	Contrast reflects the clarity of the image and the depth of the texture grooves. The deeper the texture grooves, the greater the contrast is, and the clearer the visual effect will be. On the contrary, if the contrast is small, the grooves are shallow; thus, the effect will be fuzzy.
Dissimilarity	The dissimilarity reflects the total amount of local gray changes in the image. However, different from contrast, the weight of dissimilarity increases linearly with the distance between matrix elements and diagonal.
ASM (Angular Second Moment)	ASM is used to describe the uniformity of gray image distribution and the thickness of texture. If all values of GLCM are very close, the ASM value will be smaller. If the values of matrix elements differ greatly, the ASM value will be larger.

**Table 3:** Values of hyperparameters.

Hyperparameter	Value	Hyperparameter	Value
Learning Rate	0.0001	Decay Rate	0.9000
Decay Steps	4000	Weight Decay	0.0001
End Learning Rate	0.0000	Batch Size	50

**Table 4:** Demographic characteristics.

Age Range	F	M	Age Range	F	M
[4, 5)	0	1	[14, 15)	56	38
[5, 6)	5	1	[15, 16)	52	29
[6, 7)	2	1	[16, 17)	57	28
[7, 8)	7	2	[17, 18)	68	26
[8, 9)	11	10	[18, 19)	65	21
[9, 10)	22	32	[19, 20)	17	6
[10, 11)	50	51	[20, 21)	9	0
[11, 12)	61	48	[21, 22)	1	2
[12, 13)	61	51	[22, 23)	1	0
[13, 14)	65	48	[23, 24)	0	1

Units for age range: years old; F: numbers of CBCT files of females; M: numbers of CBCT files of males.

**Table 5:** Evaluation of age range prediction model by midpalatal suture image features.

	Evaluation Parameters				
Label (Age Range)	AUC	Precision	Recall	F1-Score	Test Sample
0 (4–10 years old)	0.9106	0.5926	0.8000	0.6809	20
1 (11–12 years old)	0.6825	0.4348	0.5000	0.4651	20
2 (13–14 years old)	0.6581	0.6923	0.4500	0.5455	20
3 (15–16 years old)	0.7262	0.6000	0.6000	0.6000	20
4 (17–23 years old)	0.7887	0.5882	0.5000	0.5405	20
Total test sample	100				
Average AUC	0.7532				

## Data Availability

Not applicable.
